# Detailed Distribution of Lipids in Greenshell™ Mussel (*Perna canaliculus*)

**DOI:** 10.3390/nu6041454

**Published:** 2014-04-11

**Authors:** Matthew R. Miller, Luke Pearce, Bodhi I. Bettjeman

**Affiliations:** The New Zealand Institute for Plant & Food Research Limited, PO Box 5114, Nelson 7010, New Zealand; E-Mails: Luke.pearce@plantandfood.co.nz (L.P.); bodhi.bettjeman@plantandfood.co.nz (B.I.B.)

**Keywords:** phospholipids, triacylglycerols, fatty acid composition, eicosapentaenoic acid, docosahexaenoic acid

## Abstract

Greenshell™ mussels (GSM–*Perna canaliculus*) are a source of omega-3 (*n-*3) long-chain polyunsaturated fatty acids (LC-PUFA). Farmed GSM are considered to be a sustainable source of LC-PUFA as they require no dietary inputs, gaining all of their oil by filter-feeding microorganisms from sea water. GSM oil is a high-value product, with a value as much as 1000 times that of fish oils. GSM oil has important health benefits, for example, anti-inflammatory activity. It also contains several minor lipid components that are not present in most fish oil products, and that have their own beneficial effects on human health. We have shown the lipid content of the female GSM (1.9 g/100 g ww) was significantly greater than that of the male (1.4 g/100 g ww). Compared with male GSM, female GSM contained more *n-*3 LC-PUFA, and stored a greater proportion of total lipid in the gonad and mantle. The higher lipid content in the female than the male GSM is most likely related to gamete production. This information will be useful to optimize extraction of oils from GSM, a local and sustainable source of *n-*3 LC-PUFA.

## 1. Introduction

The New Zealand Greenshell™ mussel (GSM), *Perna canaliculus*, is indigenous to the coastlines of New Zealand. GSM are distinguishable by the green coloration of the shell near the lip, which gives the mussel its name. Since the establishment of GSM aquaculture in New Zealand in 1969 [1], the GSM industry has seen consistent and substantial growth. In 2011, the export value of GSM was 218 million New Zealand dollars (increased from approximately 40 million NZ dollars in 1989) from a product volume of more than 38,000 t (~90,000 t at harvest) [2]. GSM are sold as food, and are also used to produce high-value nutraceuticals including oil extracts and freeze-dried mussel powders, for example, Lyprinol^®^ and Seatone^®^. Studies have shown that lipid extracted from GSM has numerous health benefits, including the ability to reduce inflammation [3,4,5,6,7,8,9,10,11,12,13]. GSM lipid contains a high proportion of omega-3 long-chain (C ≥ 20) polyunsaturated fatty acids (*n-*3 LC-PUFA), predominantly docosahexaenoic acid (DHA, 22:6*n-*3) and eicosapentaenoic acid (EPA, 20:5*n-*3), which are split between the triacyglycerol and polar lipid classes [14]. There are also several minor lipid components in GSM oil, including non-methylene-interrupted (NMI)-FA, plasmalogen, phytosterols, and furan fatty acids [14,15]. These minor lipids are not present in most fish oil products, and some have been shown to have beneficial effects on human health [15,16,17,18]. Because of the low concentration of oil in GSM [14,19] and the expensive extraction techniques (supercritical CO_2_ with ethanol as a co-solvent, or chemical extraction) required to extract it, GSM oil is very expensive. The estimated price is NZ $3000/kg.

There has been extensive research confirming the health benefits of increased consumption of *n-*3 LC-PUFA, especially DHA and EPA [20,21,22,23]. Consequently, there is an increasing market for *n-*3 marine oil supplements. The proportion of *n-*3 LC-PUFA is higher in GSM oil [14] than in certain fish oils, such as those extracted from sardine, anchovy, and cod liver. GSM obtain *n-*3 LC-PUFA from their diet, which is rich in zooplankton and phytoplankton [24,25]. GSM are considered to be one of the most sustainable sources of *n-*3 LC-PUFA as they are farmed, rather than wild harvested, and do not require any dietary inputs for their nutrition. The lipid content and FA profile of GSM have been analyzed previously [14,19,26,27]. Those studies reported that polar lipids (PL) were the major lipid class (42.5–61.7 g/100 g) in GSM, followed by triacylglycerides (TAG) (17.8–49.2 g/100 g) with the remainder made up of sterols (5.5–6.8 g/100 g), free fatty acids (FFA) (2.9–14.9 g/100 g), and trace amounts of wax esters [14,19]. Previous studies reported that the concentration of PUFA in GSM lipid ranged from 19 to 49.1 g/100 g, with 6–12 g/100 g DHA, and 8–24 g/100 g EPA [14,28]. The lipid content of GSM, and the lipid classes and FA profile of GSM oil, are affected by many factors, including the season, location, and the types and amounts of algae consumed. To date, there have been no reports on the FA profiles of lipid extracted from different organs of the GSM. 

In this study, we investigated the lipid classes and fatty acid profile of GSM lipids, and analyzed the lipid content of male and female GSM. To analyze lipid localization within the GSM, we dissected the GSM body into five components: the mantle, gonad, heart and foot, posterior adductor muscle, and the digestive gland. A knowledge of the differences in the amounts and composition of lipids between genders, and of how physiological function affects GSM lipids, will be useful to optimize the lipid extraction process, which would benefit the GSM marine oil industry. 

## 2. Experimental Section

### 2.1. Sampling

**Figure 1:**
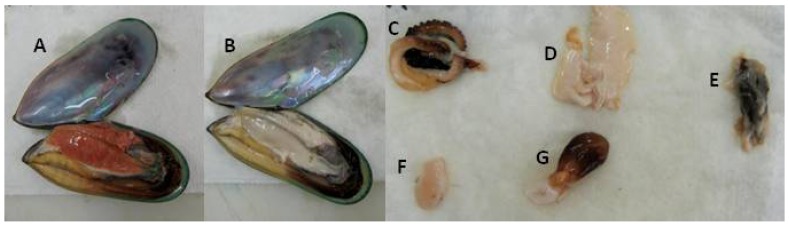
(**A**) Female Greenshell™ mussel (*Perna canaliculus*) with orange gonad; (**B**) Male Greenshell™ mussel with creamy white gonad. Dissected organs of a male Greenshell™ mussel: (**C**) mantle; (**D**) gonad; (**E**) digestive gland and digestive gland; (**F**) posterior adductor muscle; (**G**) heart and foot.

Whole live GSM were collected 12 November 2012 from the Marlborough Sounds (South Island, New Zealand) and stored in a recirculating sea water system until experiments began on 16 November 2012. After transport and dry storage, mussels were rehydrated for 24 h in 100 L bins supplied with 10 L/min filtered seawater on a flow-through basis with auxiliary aeration. The mussels were weighed, shucked, and then the mussel meat was weighed. 10 Female and 10 male mussels were selected for lipid extraction based on gonad color; gonads of males are creamy white while those of females are various shades of orange (Figure 1). The data for one mussel was excluded from the analysis because that individual was an outlier in terms of size and oil content. Six female and six male mussels were used for analyses of the various components of the GSM body. The shell and meat were weighed and then the meat was dissected into the mantle, gonad, posterior adductor muscle, heart/foot, and the digestive gland (Figure 1). The gonad was not able to be completely separated, and some gonad tissue remained attached to the digestive gland. Other un-dissectible smaller organs including the gill and labial palp were omitted from this analysis. All samples were stored at −80 °C prior to freeze drying before lipid extraction.

The mussels were assigned a condition index as follows:

condition index (CI) = dry meat weight/(whole weight-shell weight) × 100 [29]
(1)

### 2.2. Lipid Extraction, Fractionation, and Fatty Acid Analysis

GSM oils were extracted using a modified Bligh and Dyer protocol [30]. A single phase extraction with CHCl_3_:MeOH:H_2_O (1:1:0.9, v/v/v) yielded the total lipid extract (TLE). Lipid classes were analyzed with an Iatroscan MK V thin*-*layer chromatography-flame ionization detector (TLC-FID) (Iatron Laboratories, Tokyo, Japan). Samples were spotted onto silica gel SIII Chromarods (5-µm particle size) and developed in a glass tank lined with pre-soaked filter paper. The solvent system used for lipid separation was hexane: diethyl ether: acetic acid (60:17:0.1, v/v/v). After development for 25 min, the Chromarods were oven*-*dried and analyzed immediately to minimize adsorption of atmospheric contaminants. Lipid classes were quantified using Azur v5.0 software (DATALYS, St Martin D’Heres, France). The FID was calibrated for each compound class using the following compounds: phosphatidylcholine; cholesterol; cholesteryl ester; oleic acid; hydrocarbon (squalene); wax ester (derived from fish oil); and triacylglycerol (TAG, derived from fish oil).

An aliquot of the TLE from each sample type was trans-methylated in methanol: chloroform: hydrochloric acid (10:1:1, v/v/v) for 1 h at 100 °C. After addition of water the mixture was extracted three times with hexane: chloroform (4:1, v/v) to obtain fatty acid methyl esters (FAME). Samples were completed to 1 mL with an internal injection standard (23:0 or 19:0 FAME) and analyzed by gas chromatography mass spectrometry (GC-MS). The analytical system consisted of a Shimadzu 2010 QP GC-MS equipped a Restek GTx silica capillary column (30 m × 0.25 mm i.d., 0.25 µm film thickness). Samples (1 µL) were injected via a splitless injector at 220 °C. The column temperature program was as follows: 60 °C at 0 min; 40 °C min^−1^ to 100 °C; then 10 °C min^−1^ to 170 °C; then 5 °C min^−1^ to 185 °C; 2 min hold; then 3 °C min^−1^ to 197 °C; then 0.5 °C min^−1^ to 199 °C; 1 min hold; then 5 °C min to 230 °C; 3 min hold; then 5 °C min^−1^ to 250 °C; 5 min hold. Helium was the carrier gas. GC results were typically repeatable to within ±5% of the area of each individual component in replicate analyses.

Different classes of lipids were separated using a solid phase extraction (SPE) cartridge containing 500 mg silica (Grace Pure, Grace Davison Discovery Sciences, North Shore City, New Zealand). The SPE column was pre-treated with chloroform, and then a TLE sample from whole mussel, mantle, gonad, or digestive gland samples was loaded onto the column. Lipids were eluted with 10 mL chloroform followed by 10 mL methanol [31]. The oil fractions were concentrated by rotary evaporation and added to vials for analysis. Success of elution was confirmed via thin*-*layer chromatography (TLC).

### 2.3. Sterol Analysis

Sterols were isolated by saponifying 200 µL TLE in 5% w/v KOH in methanol/water (8:2 v/v) at 60 °C for 3 h. The mixture was cooled to room temperature and then 1 mL water was added. The sterols were then extracted twice using 1 mL hexane/chloroform (4:1). The extracted total non*-*saponifiable neutral (TSN) lipid fraction was transferred to labeled vials for further analysis. The TSN lipid fraction was blown down under a nitrogen stream and then heated at 60 °C with 50 µL *N*,*O*-bis(trimethylsilyl)trifluoroacetamide (BSTFA) for 40 min. BSFTA was evaporated under nitrogen and the sample was made up to 2 mL with chloroform before GC-MS analysis. Samples were injected into the GC at an oven temperature of 100 °C, which was increased to 300 °C at a rate of 10 °C/min and then held for 30 min. The mobile phase carrier gas was He. The silylated sterols were identified by their retention times and characteristic peaks in the mass spectrum [32].

### 2.4. Statistical Analysis

Mean values and standard error are reported. Normality and homogeneity of variance were confirmed and mean values were compared by one-way analysis of variance (ANOVA). Multiple comparisons were conducted using the Tukey-Kramer HSD (honestly significant difference) test. Differences were considered significant at *p* ≤ 0.05. Statistical analyses were performed using GenStat 14th Edition software.

## 3. Results

### 3.1. Lipid Content of Whole GSM

The lipid content of whole female GSM (1.9 g/100 g ww; 9.3 g/100 g dw) was significantly (*p <* 0.01) higher than that of male GSM (1.4 g/100 g ww; 7.4 g/100 g dw). The average total meat mass per individual was 18.3 g ± 3.3 g for females and 19.87 g ± 2.2 g for males. The Condition Index (CI) was 11.25 ± 2.0 for females and 9.57 ± 1.6 for males. There was no statistically significant difference between the two genders in the CI, size, or weight. 

### 3.2. Lipid Classes of GSM

The major lipid class in both genders of GSM was PL. There was a significantly (*p <* 0.03) higher concentration of PL in males (82.3 g/100 g TLE) than in females (77.8 g/100 g TLE) (Table 1). The concentration of TAG in females (19.4 g/100 g TLE) was significantly higher (*p <* 0.03) than that in males (13.2 g/100 g TLE). The minor lipid classes in males and females were sterols (2.8–2.9 g/100 g TLE) and trace amounts (0.1 g/100 g TLE) of free fatty acids (FFA).

**Table 1:** Absolute FA (wet weight) and proportion of lipid classes (g/100 g TLE) in female and male Greenshell™ mussel (GSM) oil.

Fatty acids (mg/g mussel ww)	Female	Male	F	*p*
14:0	10.6 ± 2.1	7.3 ± 1.2	18.2	<0.001
16:0	47.3 ± 6.9	42.2 ± 6.1		
17:0	2.4 ± 0.4	2.6 ± 0.4		
18:0	11.7 ± 1.8	12.1 ± 2.0		
Other SFA	5.6 ± 1.1	5.4 ± 0.7		
Total SFA	77.7 ± 12.0	69.5 ± 9.6		
16:1*n-*7	18.3 ± 3.1	12.0 ± 2.0	26.5	<0.001
18:1*n-*9	3.0 ± 0.6	2.8 ± 0.7		
18:1*n-*7	7.6 ± 1.5	5.9 ± 1.0	7.7	<0.05
20:1*n-*9	9.8 ± 1.5	10.0 ± 2.1		
20:1*n-*7	4.3 ± 1.5	3.0 ± 0.6	5.9	<0.05
Other MUFA	1.7 ± 0.5	0.9 ± 0.3	15.5	<0.001
Total MUFA	44.7 ± 7.4	34.6 ± 5.1	11.4	<0.01
18:4*n-*3	5.7 ± 1.0	3.2 ± 0.7	37.8	<0.001
18:2*n-*6	3.7 ± 0.7	3.1 ± 0.6		
18:3*n-*3	2.7 ± 0.3	2.0 ± 0.5	14.1	<0.01
20:4*n-*6	4.6 ± 0.7	4.5 ± 1.0		
20:5*n-*3	60.9 ± 11.4	42.5 ± 6.3	18.0	<0.001
20:2	5.3 ± 0.7	5.8 ± 1.8		
20:2NMI	3.2 ± 0.5	2.7 ± 0.5		
22:6*n-*3	59.2 ± 10.2	52.9 ± 8.1		
22:5*n-*3	4.1 ± 0.7	3.2 ± 0.5	10.05	<0.01
22:2NMI	5.0 ± 0.8	4.7 ± 1.1		
Other PUFA	7.7 ± 1.5	7.6 ± 2.1		
Total PUFA	162.1 ± 25.4	132.2 ± 19.3	7.9	<0.01
Total *n-*3	132.8 ± 22.5	104.3 ± 14.5	10.2	<0.01
Total *n-*6	12.0 ± 1.7	11.1 ± 2.2		
Total other	9.7 ± 6.4	10.1 ± 7.8		
**Lipid Classes (g/100 g TLE)**				
TAG	19.4 ± 4.9	13.8 ± 3.2	5.8	0.03
ST	2.8 ± 1.3	2.9 ± 0.5		
FFA	tr	tr		
PL	77.8 ± 4.5	82.3 ± 3.2	6.0	0.03
**Lipid content**				
g lipid /100 g wet weight	1.9 ± 0.2	1.4 ± 0.3	14.7	0.001
g lipid/100 g dry weight	9.2 ± 1.1	7.4 ± 1.5	8.7	0.01

Values are mean ± standard error (*n* = 9). ww; wet weight; Other SFA: Sum of 15:0, 17:0, 20:0, 22:0 and 24:0; Other MUFA: Sum of 16:1*n-*5, 18:1*n-*7trans, 18:1*n-*5, 22:*n-*11 and 22:1*n-*9; Other PUFA: Sum of 16:2*n-*6, 18:3*n-*6, 20:4*n-*6, 20:2*n-*6, 22:5*n-*6, 22:4*n-*6 and 22:2*n-*6; Other includes fatty aldehydes and 4,8,12 trimethyl tetradecanoic acid (4,8,12-TMTD); NMI, non-methylene interrupted; TAG; triacylglycerols; PL: polar lipid; ST: sterols, FFA; free fatty acids; tr: trace.

### 3.3. FA Profiles of GSM

The major FA class (in % FA) was PUFA (53.8–55.9 g/100 g TLE), the main component of which was *n-*3 LC-PUFA (42.4–45.6 g/100 g TLE). The *n-*3/*n-*6 ratio was 6.6 in male GSM and 8.3 in female GSM. The major fatty acids (>10 g/100 g TLE) in both male and female GSM were EPA, DHA, and palmitic acid (16:0 PA). There were small amounts of NMI FA, including 20:2 NMI FA (2.8–3.4 g/100 g TLE) and 22:2 NMI FA (1.0–1.9 g/100 g TLE). The absolute FA content of the male and female (in mg/g ww) are shown in Table 1. Compared with male GSM, female GSM showed significantly (*p <* 0.01) higher concentrations of alpha-linolenic acid (ALA, 18:3*n-*3), stearidonic acid (SDA 18:4*n-*3), EPA, docosapentaenoic acid (DPA(*n-*3), 22:5*n-*3), total *n-*3s, and total PUFA. 

### 3.4. Lipid Content and Lipid Classes in Different Organs

**Figure 2:**
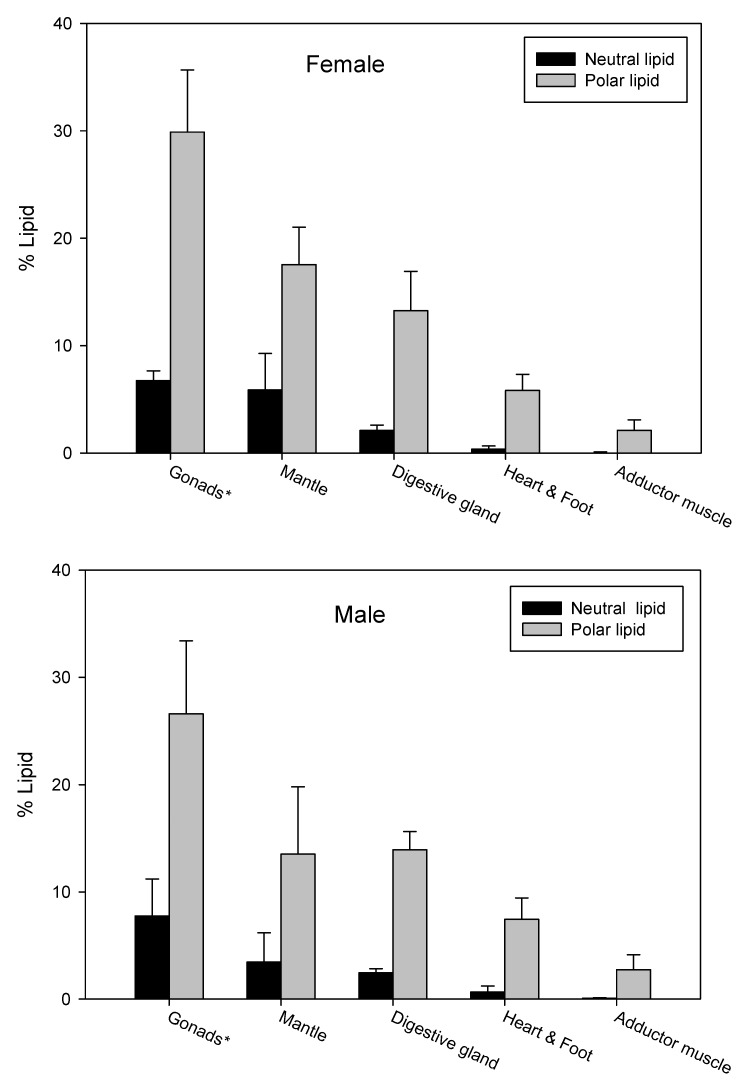
Lipid distribution in female and male Greenshell™ mussels (GSM) organs. * Gonad also contained gill and labial palp. Values are mean ± standard error (*n* = 9).

We used SPE to separate the non*-*polar and polar lipid fraction of TLE from the whole male and female GSM and from the various organs (Figure 2). The success of chromatographic separation was confirmed by TLC-FID. The most lipid-rich organs were the gonad (male, 3.1 g lipid/100 g ww; female, 4.0 g lipid/100 g ww), digestive gland (male, 3.1 g lipid/100 g ww; female, 3.7 g lipid/100 g ww) and mantle (1.5 g lipid/100 g ww; female, 2.0 g lipid/100 g ww). There were lower concentrations of lipid in the heart/foot (male, 1.3 g lipid/100 g ww; female, 1.0 g lipid/100 g ww) and adductor muscle (male, 1.2 g lipid/100 g ww; female, 0.8 g lipid/100 g ww) (Table 2, Supplementary Table S1). In all organs, PL was the major lipid class in the TLE. There were higher concentrations of TAG in the digestive gland (male, 28.8 g/100 g TLE; female, 33.5 g/100 g TLE) and gonad (male, 38.5 g/100 g TLE; female, 33.7 g/100 g TLE) than in the other organs. The lowest concentrations of TAG were in the heart/ foot and adductor muscle samples (<8 g/100 g TLE). The main lipid class in the TLE from those samples was PL (>80 g/100 g TLE).

### 3.5. FA Profiles of Lipids from Different Organs

In both male and female GSM, there were no differences in the proportions of the major FA (those detected at more than 10 g/100 g TLE; *i.e*., EPA, DHA, and PA) among the five different organs. For the minor FA, the proportions of SDA and ALA in the TLE from the digestive gland were significantly (*p <* 0.01) greater in the female GSM than the male GSM.

### 3.6. Sterols

Saponification of the TLE produced a total non*-*saponifiable neutral (TSN) fraction, which contained mainly sterols (Table 3). GC-MS analysis was performed after silylation of the TSN fraction. In total, 18 sterols were identified in GSM, with cholesterol being the predominant sterol. There was a significantly (*p <* 0.01) higher proportion cholesterol (30.2% of total sterols) in male GSM than in female GSM (28.5% of total sterols). The second most abundant sterol was brassicasterol; the proportion of brassicasterol was significantly (*p <* 0.01) higher in female (23.4% of total sterols) than in male GSM (21.05% of total sterols). Other major sterols were 24-nordehydrocholesterol, occelasterol, trans-22-dehydrocholesterol, and 24-methlenecholesterol.

## 4. Discussion

### 4.1. Male vs. Female GSM

In this study the lipid content of female GSM was significantly (*p <* 0.01) higher than that of male GSM even though it was previously reported that female mussels contain lower levels of total lipids [33]. Also, there was a significantly (*p <* 0.01) higher concentration of the TAG lipid class in the female than in the male. Because female GSM contained larger amounts of lipid than did males, the total amount of FA was higher in females than in males. In particular, the amounts of FA in the *n-*3 LC-PUFA class (ALA, SDA, EPA, and DPA) were all significantly (*p <* 0.01) higher in female than in male GSM. Differences in the lipid profile between male and female GSM have not been published previously, although a recent report showed similar results for the mussel species *Mytilus galloprovincialis* [34]. In another study, the FA composition of male and female GSM from two locations (Marlborough Sounds and Stewart Island) was profiled over three seasons (winter, spring, summer) but the types of lipids, that is, the lipid classes, were not reported [26]. Further, it was reported that GSM contained higher levels of lipids in summer and autumn than in winter and spring, but that the highest *n-*3 content was in winter [28]. In our study, we analyzed GSM collected in late spring (November) of 2012. Our results showed differences in lipid content, lipid classes, and total FA content between male and female GSM. In future research, it would be interesting to analyze changes in lipid content, lipid class composition, and the FA profile of male and female GSM on a finer time scale. 

**Table 2:** Concentrations of fatty acids in oil extracted from mantle, gonad, and digestive gland of Greenshell™ mussels.

**Fatty acids ** **(g/100 g TLE)**	**Mantle**						**Gonad**						**Digestive gland**					***f***		***p***
	**Female**			**Male**			**Female**			**Male**			**Female**			**Male**				
14:0	2.8 ± 0.2			3.4 ± 2.2			3.5 ± 0.3			2.9 ± 0.4			3.4 ± 0.7			3.0 ± 0.4				
16:0	15.5 ± 0.4			17.1 ± 4.3			16.5 ± 1.1			16.8 ± 1.5			17.1 ± 3.8			15.6 ± 1.3				
18:0	4.3 ± 0.4			4.5 ± 0.4			4.2 ± 0.3			5.2 ± 1.5			5.1 ± 1.6			4.8 ± 0.4				
Other SFA	1.8 ± 0.2 ^ab^			1.8 ± 0.1 ^ab^			1.7 ± 0.2 ^a^			1.7 ± 0.1 ^a^			1.7 ± 0.2 ^ab^			2.4 ± 0.9 ^b^		2.9		0.03
Total SFA	25.3 ± 0.4			27.7 ± 6.6			26.6 ± 1.4			27.5 ± 2.6			28.1 ± 6.3			26.5 ± 1.3				
16:1*n-*7	5.2 ± 0.6 ^ab^			5.3 ± 1.8 ^ab^			6.3 ± 0.4 ^ab^			4.7 ± 1.6 ^a^			6.8 ± 0.6 ^b^			6.2 ± 0.8 ^ab^		3.2		0.02
18:1*n-*9	1.3 ± 0.5 ^ab^			0.9 ± 0.2 ^a^			1.1 ± 0.3 ^ab^			1.0 ± 0.2 ^ab^			1.7 ± 0.6 ^b^			1.4 ± 0.3 ^ab^		3.0		0.03
18:1*n-*7	2.2 ± 0.3 ^ab^			2.0 ± 0.2 ^a^			2.7 ± 0.2 ^c^			2.6 ± 0.4 ^ab^			2.5 ± 0.2 ^bc^			2.4 ± 0.2 ^abc^		6.2		<0.001
20:1*n-*9	4.2 ± 0.4			4.2 ± 0.7			3.5 ± 0.3			3.5 ± 0.6			3.8 ± 0.5			3.7 ± 0.3				
20:1*n-*7	1.3 ± 0.1			1.0 ± 0.4			1.3 ± 0.1			1.2 ± 0.3			1.5 ± 0.4			1.2 ± 0.2				
Other MUFA	0.5 ± 0.2 ^ab^			1.9 ± 1.3 ^b^			0.5 ± 0.1 ^ab^			0.3 ± 0.1 ^a^			0.9 ± 0.8 ^ab^			1.6 ± 1.6 ^ab^		3.1		0.02
Total MUFA	14.6 ± 0.9 ^ab^			15.3 ± 1.0 ^bc^			15.3 ± 0.8 ^bc^			13.3 ± 1.2 ^a^			17.1 ± 1.3 ^c^			16.4 ± 1.2 ^bc^		9.4		<0.001
18:4*n-*3	1.2 ± 0.4 ^ab^			1.0 ± 0.3 ^a^			2.0 ± 0.4 ^bc^			1.4 ± 0.6 ^abc^			2.1 ± 0.6 ^c^			2.1 ± 0.4 ^c^		6.4		<0.001
18:2*n-*6	1.0 ± 0.2 ^a^			1.0 ± 0.1 ^a^			1.3 ± 0.3 ^ab^			1.1 ± 0.2 ^a^			1.5 ± 0.3 ^b^			1.3 ± 0.2 ^ab^		4.5		0.01
18:3*n-*3	0.6 ± 0.2 ^a^			0.7 ± 0.2 ^ab^			1.0 ± 0.2 ^ab^			0.8 ± 0.3 ^ab^			1.0 ± 0.2 ^b^			1.0 ± 0.1 ^b^		4.4		0.01
20:4*n-*6	1.9 ± 0.2 ^b^			1.9 ± 0.5 ^b^			1.3 ± 0.1 ^a^			1.2 ± 0.1 ^a^			1.3 ± 0.2 ^a^			1.3 ± 0.2 ^a^		9.3		<0.001
20:5*n-*3	18.9 ± 1.5			17.9 ± 1.5			22.0 ± 1.5			19.5 ± 3.7			18.1 ± 4.0			17.6 ± 3.0				
20:2NMI	2.9 ± 0.7			3.0 ± 0.9			1.6 ± 0.2			1.2 ± 0.2			1.9 ± 0.3			1.7 ± 0.2				
20:4*n-*6	0.4 ± 0.1			0.3 ± 0.1			0.2 ± 0.1			0.2 ± 0.1			1.3 ± 0.2			1.3 ± 0.3				
20:2*n-*6	1.5 ± 0.3 ^c^			1.4 ± 0.3 ^bc^			0.8 ± 0.1 ^a^			0.7 ± 0.1 ^a^			1.0 ± 0.1 ^ab^			0.9 ± 0.1 ^a^		14.8		<0.001
22:6*n-*3	22.0 ± 1.4			20.5 ± 4.0			19.2 ± 1.3			21.9 ± 2.4			17.9 ± 3.8			19.1 ± 1.5				
22:5*n-*3	1.5 ± 0.3			1.3 ± 0.4			1.2 ± 0.1			2.0 ± 2.1			1.4 ± 0.3			1.1 ± 0.1				
22:2NMI	2.2 ± 0.4 ^b^			1.9 ± 1.0 ^ab^			1.2 ± 0.1 ^a^			1.2 ± 0.2 ^a^			1.7 ± 0.3 ^ab^			1.6 ± 0.3 ^ab^		4.4		0.01
Other PUFA	1.8 ± 0.3 ^ab^			1.8 ± 0.5^a^			2.9 ± 2.0 ^ab^			2.6 ± 1.3 ^ab^			3.3 ± 1.4 ^ab^			4.4 ± 2.2 ^b^		2.7		0.04
Total PUFA	56.0 ± 2.3			52.8 ± 7.1			54.0 ± 1.8			54.5 ± 2.5			51.6 ± 7.1			52.5 ± 2.5				
Total *n-*3	44.2 ± 1.6			41.5 ± 4.5			45.4 ± 1.9			45.7 ± 4.1			40.7 ± 8.1			41.2 ± 4.0				
Total *n-*6	5.5 ± 0.5			5.2 ± 1.0			4.8 ± 0.8			4.9 ± 0.9			6.0 ± 1.1			6.7 ± 1.8				
Total other	3.9 ± 1.8			3.8 ± 1.4			2.9 ± 2.1			4.4 ± 1.5			2.8 ± 1.1			4.2 ± 1.7				
**Lipids class ** **(g/100 g)**																				
TAG	22.9 ± 8.0 ^ab^			18.4 ± 6.0 ^a^			33.7 ± 4.3 ^ab^			38.5 ± 17.6 ^b^			33.5 ± 10.8 ^ab^			28.8 ± 8.1 ^ab^		3.3		0.02
ST	3.5 ± 0.9			3.2 ± 0.4			1.2 ± 0.6			6.1 ± 11.0			1.0 ± 1.1			1.7 ± 0.6				
PL	73.5 ± 7.4 ^b^			77.3 ± 6.4 ^b^			64.5 ± 4.0 ^ab^			54.6 ± 12.7 ^a^			65.1 ± 10.6 ^ab^			68.7 ± 7.6 ^ab^		5.1		0.002
**Lipid content**																				
g lipid /100 g wet weight	2.0 ± 0.4 ^a^			1.5 ± 0.7 ^a^			4.0 ± 0.6 ^b^			3.1 ± 0.6 ^b^			3.7 ± 0.3 ^b^			3.1 ± 0.3 ^b^		19.8		<0.001
g lipid/100 g dry weight	11.8 ± 1.8 ^b^			7.9 ± 3.6 ^a^			13.3 ± 1.4 ^b^			11.7 ± 2.0 ^b^			13.2 ± 1.0 ^b^			11.9 ± 1.1 ^b^		5.6		<0.001

TLE: Total lipid extract. Values are mean ± standard error (*n* = 6). Other SFA: Sum of 15:0, 17:0, 20:0, 22:0, and 24:0. Other MUFA: Sum of 16:1*n-*5, 18:1*n-*7trans, 18:1*n-*5, 22:1*n-*11, and 22:1*n-*9. Other PUFA: Sum of 16:2*n-*6, 18:3*n-*6, 20:4*n-*6, 20:2*n-*6, 22:5*n-*6, 22:4*n-*6, and 22:2*n-*6. Other includes fatty aldehydes and 4,8,12 trimethyl tetradecanoic acid (4,8,12-TMTD). TAG: triacylglycerols, PL: polar lipid; ST: sterols.

**Table 3:** Sterol content of female and male Greenshell™ mussel (GSM) oil extract.

Sterols g/100 g total sterols	Sterol name	Female	Male	f	*p*
24-nordehydrocholesterol	24-nordecholesta-5,22*E*-dien-3β-ol	6.3 ± 0.9	6.7 ± 0.4		
24-nordehydrocholestanol	24-nor-5α-cholest-22*E*-en-3β-ol	0.1 ± 0.0	0.2 ± 0.0		
Occelasterol	27-nor-24-methylcholest-5,22*E*-dien-3β-ol	4.3 ± 0.3	4.5 ± 0.3		
Trans-22-dehydrocholesterol	Cholesta-5,22*E*-dien-3β-ol	12.4 ± 0.7	13.1 ± 0.8		
Trans-22-dehydrocholestanol	5α-cholesta-22*E*-en-3β-ol	0.3 ± 0.1	0.2 ± 0.1		
Cholesterol	Cholest-5-en-3β-ol	28.5 ± 1.5	30.2 ± 0.8	11.1	0.004
Cholestanol	5α-cholestan-3β-ol	0.3 ± 0.2	0.5 ± 0.4		
Brassicasterol	24-methylcholesta-5,22*E*-diene-3β-ol	23.4 ± 0.9	21.0 ± 1.4	21.3	<0.001
Brassicastanol	24-methyl-5α-cholest-22*E*-en-3β-ol	0.3 ± 0.3	0.3 ± 0.2		
Ergosterol	24-methylcholesta-5,7,22*E*-triene-3β-ol	0.6 ± 0.4	0.0 ± 0.1	16.6	<0.001
24-Methylenecholesterol	24-methylcholesta-5,24(28)-en-3β-ol	16.2 ± 1.2	16.6 ± 1.6		
24-Methylcholesterol	4-methyl-5α-cholestan-3β-ol	1.9 ± 0.4	1.6 ± 0.7		
Stigmastanol/Porifasterol	24-ethyl-5α-cholesta-5,22*E*-diene-3β-ol	0.6 ± 0.2	0.7 ± 0.2		
Sitosterol	24-ethyl-5α-cholestan-3β-ol	1.8 ± 0.4	2.1 ± 0.3		
Isofucosterol	24-ethylcholesta-5,24(28)*Z*-dien-3β-ol	1.7 ± 0.4	1.7 ± 0.4		
Unknown sterols		1.3 ± 0.1	0.3 ± 0.1	40.8	<0.001

Values are mean ± standard error (*n* = 9); Unknown sterols could not be identified from MS data.

There is significant biological investment by the GSM into the production/storage of lipids during gonad development during winter, before spawning [28]. In general, the amount of lipid increases in GSM before spawning, and decreases to its lowest level immediately after spawning as a result of lipid loss during reproduction. GSM can also spawn in autumn (March–April) or shed eggs throughout their life cycle [28]. These reproductive events could explain differences in FA and lipid accumulation throughout the seasons. The higher lipid content in the female GSM is likely associated with oogenesis, during which lipid globules and small quantities of glycogen accumulate in the eggs [35]. There is a large variation in the timing and duration of gonad development in GSM and other mollusks, and the timing of gametogenesis varies according to the season and location [35]. A better understanding of the reproductive cycle, and how it affects the amount of lipid in GSM, may allow GSM oil producers to optimize the harvest time to obtain higher lipid yields.

Little is known about FA biosynthesis in GSM. In general, most bivalve species gain the majority of FA from their diet, although biochemical modifications of some FA occur in some species. The idea of “you are what you eat” has led to the study of signature lipids that can help to shed light on the prey-predator relationships in ecosystems. When analyzed using high-powered statistical models, signature FA profiles can reveal prey-predator relationships in a food web. GSM are filter feeders that consume a variety of phytoplankton and zooplankton [24,25]. Recently, studies on feeding blue mussels (*Mytilus edulis*) organic waste from an aquaculture facility showed that when they were fed on a non*-*traditional (fish waste) diet, they did not feed or grow, but instead showed significant decreases in growth and total lipid content [36,37]. Phytoplankton and zooplankton are high in *n-*3 LC-PUFA, which results in the FA profile of GSM oil being rich in EPA and DHA. The types of FA present in the food sources of GSM will depend on factors such as season, location, and temperature. The similarity in the relative proportions of FA (g/100 g TLE) in males and females (and even among organs) suggested that there was little adaption of FA from their shared diet by either gender. However, the larger amount of lipid in the female GSM suggests that it has a greater capacity than the male GSM to store, and possibly biosynthesize, FA, especially *n-*3 LC-PUFA.

The biosynthetic pathway for *n-*3 LC-PUFA is unknown in GSM. Vertebrates have a poor capacity to produce DHA from precursors via the Sprecher pathway [38]. However, there is some evidence that invertebrates, such as the nematode *Caenorhabditis elegans*, have different pathways to produce *n-*3 LC-PUFA [39]. The FA results confirmed that the major biosynthetic precursors in the *n-*3 LC-PUFA pathway are present in the FA profile of GSM. These biosynthetic precursors include ALA, SDA, EPA, and DPA (*n-*3), with DHA being the final product. All of these FA except for DHA were present in significantly (*p <* 0.01) higher proportions of total FA in the female than in the male GSM. The higher concentrations of ALA, SDA, EPA, and DPA (*n-*3) may indicate that the female GSM has a greater *n-*3 LC-PUFA biosynthetic capacity during gametogenesis. Furthermore, in the digestive gland, the proportions of ALA and SDA in the FA profile were significantly (*p <* 0.01) higher in female than in male GSM. This may indicate a higher rate of FA biosynthesis in the digestive gland before lipids are transported for storage in the gonad. However, these FA results could also indicate that female GSM have an enhanced capacity to filter, process, and store *n-*3 LC-PUFA. To date, there have been no published reports of feeding trials to clarify aspects of lipid metabolism and/or storage in GSM. Therefore, it remains a matter of speculation whether the higher concentrations of FA in female than in male GSM are a result of enhanced biosynthesis or greater storage capacity.

Minor bioactive FA present in the GSM profiles included NMI PUFA, 4,8,12 trimethyl tetradecanoic acid (4,8,12-TMTD), and fatty aldehydes, all of which have been reported previously [14,19]. NMI FA are present in various mollusks at concentrations of up to 20% of wet weight [16]. Mollusks can synthesize NMI, which are believed to have structural and functional roles in biological membranes [16], via biosynthetic pathways that are still not fully understood. Our results showed that there were differences in the amount of NMI between male and female GSM, and that there was significantly less NMI in the gonad than in the other organs. Fatty aldehydes and 4,8,12-TMTD were present in GSM, but at very low concentrations (<1 g/100 g TLE), and were not included in the FA profiles. The amounts of both 4,8,12-TMTD and fatty aldehydes were substantially lower than those detected in our previous analyses of GSM that were collected from a similar location but at a slightly earlier time of the year (August and September 2009) [14]. Both NMI and 4,8,12-TMTD are possible indicators of red algae and/or some zooplanktonic pteropods, which could have been directly or indirectly consumed [19]. However, in other mollusks such as the green abalone, *Haliotis fulgens*, there is some evidence that 20:2 and 22:2 NMI may be metabolic products of desaturation of LC-MUFA [40]. Therefore, their presence may indicate an endogenous biosynthetic capacity of GSM.

### 4.2. Differences among Organs

Although the gonad contained the highest concentrations of lipid, the digestive gland also contained high lipid concentrations, which may be indicative of lipid-rich stomach contents. The lipid extracted from the digestive gland was darker and stickier than that extracted from the other organs. The dark color may represent non*-*enzymatic browning during extraction; this reaction occurs between oxidized lipids and primary amine groups, and has been observed in PL emulsions [41]. The digestive gland will likely contain the highest concentrations of endogenous lipases and low pH which may lead to the oxidization of lipid because of the more extreme conditions. Further, the dark color could be due to pigment residues derived from algae from the GSM diet

Generally, different lipid classes perform different functions in biological systems. Neutral lipids (*i.e*., TAG) are used as energy storage, while PL are mainly components of structural and functional parts of the cell. The lipid content of bivalves is directly linked to the gametogenic cycle, as TAGs and PLs play important roles as structural components and energy reserves in the gonads and gametes and during embryonic development [35]. Previous studies have shown the accumulation of neutral lipid reserves in eggs for a number of bivalve species, although studies looking at lipid production in GSM are not as detailed with regards to the effects of the gametogenic cycle [26,28]. It has been suggested that the digestive gland plays an important role in storing metabolic reserves, such as lipids, for use in gametogenesis and during periods of stress [35,42]. As previously mentioned, the higher proportion of *n-*3 LC-PUFA biosynthetic precursors (ALA and SDA) in the GSM digestive gland indicates that this organ is the most likely site of FA biosynthesis, and that these FA may have structural and functional roles in gamete development. The GSM analyzed in this study were collected in single sampling during a period of gametic growth; therefore, we cannot comment on changes in lipid composition during the year.

The mantle was the largest of the organs analyzed in this study, and accounts for approximately 20% of the wet weight of the mussel (Figure 2). The mantle is an important store of lipids in GSM; our results showed that this organ contained 21.5% (male mussels) and 28.0% (female mussels) of the total lipid. The main lipid class in the mantle was PL (73.5 g/100 g TLE in females; 77.3 g/100 g TLE in males) because of the large amount of structural tissue in this organ. The mantle consists of vascular connective tissue and plays a role in directing particles to the gills and deflecting other materials [35]. Our results suggested that the mantle of GSM also functions in lipid storage. The proportion of neutral lipids, which are generally used for storage, was higher in the mantle (22.9 g/100 g TLE in females; 18.4 g/100 g TLE in males) than in organs such as the adductor muscle (<3.0 g/100 g of TLE), which have different functions.

The three major organs of the GSM that store and use lipids are the gonad, digestive gland, and mantle. Our results showed that the adductor muscle and the heart and foot contained lower concentrations of lipids (3.9 g/100 g of TLE for adductor muscle and 5.4 g/100 g of TLE for heat and foot). The lipid extracted from the adductor muscle and the heart and foot showed similar FA profiles to that of the mantle. In the TLE from both the adductor muscle and the heart and foot samples, the major lipid class was PL (83.1–91.78 g/100 g of TLE) with minor amounts of TAG (1.9–7.5 g/100 g of TLE) and sterols (4.7–8.1 g/100 g of TLE). Profiles are included in the Supplementary Files. In general, the differences in lipid classes among different organs and between genders were minimal, suggesting that the diet is the main source of these FA and has the greatest influence on FA composition.

### 4.3. Sterols

The sterols in marine invertebrates are generally derived from sterols in the diet. However, some unconventional sterols are more common in primitive invertebrates, while cholesterol is found in greater proportions in more complex organisms [43]. The sterol profile of GSM reported here (Table 3) is similar to that of the only other published GSM sterol profile [19]. In a previous study, GSM were sampled from a similar region (Marlborough Sounds) at a similar time of year (October, compared with our sampling time in early November). In that study, there were only minor differences in the sterol profile among samples collected from four different sites [19]. This finding suggested that there was little difference in the diets of GSM among sites. 

Phytosterols have wide bioactivity in humans. In particular, they are considered to be effective in lowering cholesterol, and consequently may have a preventive role against vascular disease [18,44]. Further beneficial properties of phytosterols include cancer prevention [18,45]. Phytosterols and cholesterol are likely metabolized by internal and external microorganisms into other bioactive substances. Phytosterols cannot be synthesized by humans and, therefore, can only be obtained through the diet. However, GSM may have some capacity to biosynthesize cholesterol into different phytosterols depending on their stage of the life cycle, sexual maturity, and gender [46]. As they are lipophilic substances with positive health benefits, phytosterols have been added to margarines and spreads to create “functional” foods. The amount of phytosterols derived from land sources is increasing in the marine environment as a result of land base ingredients increased used in aquafeeds. Changes from marine to terrestrial sources of protein and oil for use in aquafeeds has resulted in increased amounts and different types of phytosterols in farmed Atlantic salmon [47]. Here, we report that GSM oil contained approximately 3% phytosterols, of which about one-third was cholesterol. Larger amounts of sterols (5.5%–6.9%) have been reported previously for GSM [19]. Phytosterols such as isofucosterol and occelasterol are not common in our diets as they are not typical in terrestrial food sources, but may have novel beneficial functionalities. Isofucosterol and occelasterol are most likely derived from marine algae. In future research, it would be interesting to investigate whether novel phytosterols, along with the high content of ω3 LC-PUFA in GSM, can provide increased protection against coronary heart disease in humans.

### 4.4. Implications of Results for the GSM Industry

The major implications of this work are for the GSM oil industry, which is growing in both value and volume. A detailed understanding of the location of *n-*3 LC-PUFA in GSM may allow novel processing techniques to be established. The firmer parts of the mussel (posterior adductor muscle, heart and foot) contained very low concentrations of oil and *n-*3 LC-PUFA, and removal of these organs would reduce the amount of material processed using expensive freeze-drying and super-critical oil extraction procedures. This could potentially improve yields and benefit producers. Further, development of all-female lines of mussels would give mussel oil producers a greater source of oil and *n-*3 LC-PUFA. We estimate that oil yields could increase by as much as 35%, *n-*3 yields by 27%, and EPA yields by 43% if all-female mussels were extracted at the appropriate time of year. Highest yields could be obtained if female GSM were harvested in the peak reproductive state, since the gonad is the major storage centre for lipid. Higher investment in reproduction by the female GSM gonad may be to supply ample nutrition to offspring/eggs. The FA data suggested that female GSM have a higher biosynthetic capacity than that of males, and that they also store more lipid than do males. Compared with male GSM, female GSM contained larger amounts of the *n-*3 LC-PUFA biosynthetic precursors SDA and ALA. If both genders obtain the same FA from the shared diet, the increase in these biosynthetic precursors provides further evidence of an enhanced biosynthetic capacity in female GSM.

## 5. Conclusions

The lipid profiles of GSM were evaluated on the basis of gender and anatomy. The lipid content of the female GSM (1.9 g/100 g ww) was significantly greater than that of the male (1.4 g/100 g ww). The major lipid class in both genders was PL. Compared with male GSM, female GSM contained more *n-*3 LC-PUFA, and stored a greater proportion of total lipid in the gonad and mantle. The higher lipid content in the female than the male GSM is most likely related to gamete production. The mantle and digestive gland were other important sites for lipid storage and/or function/production. Novel bioactives, such as NMI-FA, plasmalogens, and phytosterols were identified in GSM oil. 

## Abbreviations

ANOVA: 1-way analysis of variance
ALA: alpha-linolenic acid
BSTFA: *N*,*O*-bis(trimethylsilyl)trifluoroacetamide
CI: Condition index
DHA: docosahexaenoic acid
DPA: docosapentaenoic acid
EPA: eicosapentaenoic acid
FA: fatty acid(s)
FFA: free fatty acid(s)
FALD: dimethylacetals of aliphatic aldehydes
FAME: fatty acid(s) methyl ester
GC-MS: gas chromatography mass spectrometry
GSM: Greenshell™ mussel
HSD: honestly significant difference
LC: long chain (C ≥ 20)
MUFA: monounsaturated fatty acid(s)
NMI: non-methylene interrupted
TSN: non*-*saponifiable neutral
*n*-3: Omega 3
*n*-6: Omega 6
PUFA: polyunsaturated fatty acid(s)
SDA: stearidonic acid
SFA: saturated fatty acid(s)
TAG: triacylglycerol
TLE: total lipid extract
TMTD: trimethyl tetradecanoic acid
tr: trace amounts

## Supplementary Information

**Table S1:** Fatty acid concentration (mg/g) of Greenshell™ mussels. Polar and neutral lipid fractions were separated by solid phase extraction (SPE).

Fatty acid (mg/g ww)	Female	Male	Female	Male	F	*p*
	Polar lipid	Polar lipid	Neutral lipid	Neutral lipid		
14:0	5.3 ± 0.9 ^c^	3.5 ± 1.1 ^b^	1.9 ± 0.9 ^a^	0.9 ± 0.5 ^a^	50.8	<0.001
16:0	23.2 ± 2.8 ^b^	19.8 ± 4.3 ^b^	5.9 ± 2.7 ^a^	3.0^a^ ± 1.6 ^a^	110.8	<0.001
18:0	5.6 ± 0.8 ^b^	5.8 ± 1.3 ^b^	1.0 ± 0.5 ^a^	0.7 ± 0.3 ^a^	113.0	<0.001
Other SFA	2.6 ± 0.4 ^b^	2.5 ± 0.7 ^b^	0.5 ± 0.3 ^a^	0.3 ± 0.2^a^	84.8	<0.001
Total SFA	37.9 ± 4.5 ^b^	32.8 ± 7.4 ^b^	9.5 ± 4.4 ^a^	5.2 ± 2.7 ^a^	105.7	<0.001
16:1*n-*7	9.2 ± 1.6 ^b^	5.7 ± 1.8 ^b^	3.3 ± 1.7 ^a^	1.6 ± 1.0 ^a^	45.5	<0.001
18:1*n-*9	1.5 ± 0.4 ^b^	1.3 ± 0.3 ^b^	0.6 ± 0.3 ^a^	0.4 ± 0.2 ^a^	26.6	<0.001
18:1*n-*7	3.7 ± 0.5 ^b^	2.9 ± 0.9^b^	1.1 ± 0.6 ^a^	0.6 ± 0.3 ^a^	59.5	<0.001
20:1*n-*9	4.7 ± 0.7 ^b^	4.6 ± 0.8 ^b^	0.7 ± 0.3	0.6^a^ ± 0.2^a^	223.3	<0.001
20:1*n-*7	2.0 ± 0.6 ^c^	1.4 ± 0.4 ^b^	0.5 ± 0.2 ^a^	0.3 ± 0.1 ^a^	28.5	<0.001
Other MUFA	0.8 ± 0.2 ^c^	0.4 ± 0.2 ^b^	0.3 ± 0.1 ^ab^	0.1 ± 0.1 ^a^	27.6	<0.001
Total MUFA	21.7 ± 2.8 ^c^	16.3 ± 4.0 ^b^	6.5 ± 2.9 ^a^	3.6 ± 1.8 ^a^	80.8	<0.001
18:4*n-*3	2.8 ± 0.4 ^d^	1.5 ± 0.5 ^c^	1.1 ± 0.6 ^b^	0.5 ± 0.3^a^	42.2	<0.001
18:2*n-*6	1.8 ± 0.3 ^b^	1.5 ± 0.4 ^b^	0.5 ± 0.3 ^a^	0.3 ± 0.2 ^a^	48.2	<0.001
18:3*n-*3	1.3 ± 0.3 ^c^	0.9 ± 0.2 ^b^	0.4 ± 0.3 ^a^	0.2 ± 0.1 ^a^	45.9	<0.001
20:4*n-*6	2.2 ± 0.3 ^b^	2.1 ± 0.4 ^b^	0.2 ± 0.1 ^a^	0.2 ± 0.1 ^a^	201.5	<0.001
20:5*n-*3	30.5 ± 5.2 ^c^	20.5 ± 5.7 ^b^	7.5 ± 3.2 ^a^	4.1 ± 1.9 ^a^	80.0	<0.001
20:2*n-*6	2.6 ± 0.5 ^b^	2.6 ± 0.4 ^b^	0.3 ± 0.1 ^a^	0.2 ± 0.0 ^a^	172.2	<0.001
20:2NMI	1.5 ± 0.1 ^b^	1.3 ± 0.3 ^b^	0.4 ± 0.1 ^a^	0.2 ± 0.1 ^a^	146.7	<0.001
22:5*n-*6	0.3 ± 0.1 ^b^	0.3 ± 0.1 ^b^	0.1 ± 0.1 ^a^	0.1 ± 0.0 ^a^	6.8	<0.001
22:6*n-*3	28.7 ± 3.7 ^b^	24.8 ± 4.9 ^b^	6.3 ± 3.3 ^a^	3.9 ± 1.7 ^a^	121.9	<0.001
22:5*n-*3	2.0 ± 0.2 ^c^	1.5 ± 0.4 ^b^	0.3 ± 0.2 ^a^	0.2 ± 0.1 ^a^	139.4	<0.001
22:2NMI	2.4 ± 0.4 ^b^	2.2 ± 0.4 ^b^	0.2 ± 0.1 ^a^	0.2 ± 0.0 ^a^	177.7	<0.001
Other PUFA	4.3 ± 1.0 ^b^	4.1 ± 1.4 ^b^	1.3 ± 1.8^a^	0.8 ± 0.6 ^a^	20.7	<0.001
Total PUFA	79.5 ± 9.6 ^c^	62.7 ± 14.0 ^b^	17.8 ± 7.9 ^a^	10.4 ± 4.5 ^a^	112.7	<0.001
Total *n-*3	65.5 ± 8.6 ^c^	49.5 ± 11.6 ^b^	15.6 ± 6.9 ^a^	9.0 ± 3.9 ^a^	107.5	<0.001
Total *n-*6	5.8 ± 1.1 ^b^	5.5 ± 1.7 ^b^	1.1 ± 0.6 ^a^	0.8 ± 0.3 ^a^	62.73	<0.001
Total other	4.3 ± 2.9 ^b^	3.9 ± 2.5 ^b^	0.2 ± 0.1 ^a^	0.2 ± 0.1 ^a^	11.2	<0.001

Values are mean ± standard error (*n*=9). ww; wet weight; Other SFA: Sum of 15:0, 17:0, 20:0, 22:0 and 24:0; Other MUFA: Sum of 16:1*n-*5, 18:1*n-*7trans, 18:1*n-*5, 22:*n-*11, and 22:1*n-*9; Other PUFA: Sum of 16:2*n-*6, 18:3*n-*6, 20:4*n-*6, 20:2*n-*6, 22:5*n-*6, 22:4*n-*6, and 22:2*n-*6; Other includes fatty aldehydes and 4,8,12 trimethyl tetradecanoic acid (4,8,12-TMTD).

**Table S2:** Fatty acid concentrations in oil extracted from Greenshell™ mussel (GSM) adductor muscle and heart and foot.

Fatty acids (g/100 g TLE)	Adductor Muscle		Heart & foot	
	Female	Male	Female	Male
14:0	1.9 ± 0.6	2.4 ± 0.5	2.6 ± 0.5	2.3 ± 0.7
16:0	18.6 ± 6.1	26.3 ± 10.1	19.0 ± 1.8	16.1 ± 4.1
17:0	1.4 ± 0.5	1.5 ± 0.4	1.5 ± 0.2	1.3 ± 0.2
18:0	5.6 ± 0.9	6.8 ± 2.7	5.2 ± 0.3	5.0 ± 0.4
Other SFA	1.7 ± 0.5	1.3 ± 0.5	2.6 ± 0.2	2.4 ± 0.5
Total SFA	29.2 ± 7.9	38.1 ± 13.4	30.8 ± 2.4	27.1 ± 5.3
16:1*n-*7	2.0 ± 1.2	2.2 ± 0.6	4.4 ± 0.7	4.0 ± 1.2
18:1*n-*9	0.7 ± 0.2	0.7 ± 0.3	0.8 ± 0.1	0.8 ± 0.2
18:1*n-*7	2.6 ± 0.8	2.1 ± 0.7	1.9 ± 0.2	1.9 ± 0.3
20:1*n-*9	4.3 ± 0.7	3.5 ± 0.9	4.7 ± 0.6	4.8 ± 0.3
20:1*n-*7	0.9 ± 0.1	0.6 ± 0.3	1.1 ± 0.1	1.2 ± 0.2
Other MUFA	0.4 ± 0.4	0.6 ± 1.0	0.4 ± 0.0	0.4 ± 0.1
Total MUFA	10.8 ± 1.1	9.8 ± 2.3	13.2 ± 0.7	13.2 ± 1.1
18:4*n-*3	0.8 ± 0.5	0.3 ± 0.4	0.7 ± 0.3	0.8 ± 0.2
18:2*n-*6	1.5 ± 0.5	1.1 ± 0.6	1.5 ± 0.2	1.5 ± 0.1
18:3*n-*3	0.8 ± 0.3	0.6 ± 0.4	0.8 ± 0.1	0.8 ± 0.1
20:4*n-*6	1.9 ± 0.3	1.2 ± 0.6	3.1 ± 0.4	3.2 ± 0.2
20:5*n-*3	13.5 ± 1.8	12.0 ± 1.0	11.3 ± 1.5	12.9 ± 3
20:2NMI	1.0 ± 0.3	0.5 ± 0.4	1.8 ± 0.3	1.7 ± 0.2
20:4*n-*6	0.4 ± 0.2	0.2 ± 0.2	0.5 ± 0.1	0.6 ± 3.2
20:2*n-*6	0.1 ± 0.1	0.1 ± 0.1	0.2 ± 0.0	0.2 ± 0.1
22:6*n-*3	25.3 ± 6.4	23.7 ± 10.8	19.6 ± 3.0	21.7 ± 0.2
22:5*n-*3	3.2 ± 0.9	2.4 ± 1.8	1.7 ± 0.4	1.9 ± 0.1
22:3	0.5 ± 0.1	0.3 ± 0.3	0.8 ± 0.2	0.9 ± 0.4
22:2NMI	2.1 ± 0.8	1.7 ± 1.5	2.3 ± 0.4	2.4 ± 0.2
Other PUFA	1.5 ± 0.5	1.0 ± 0.5	1.8 ± 0.2	2.2 ± 0.2
Total PUFA	55.9 ± 9.6	47.6 ± 13.0	50.5 ± 3.8	54.5 ± 6.6
Total *n-*3	43.6 ± 8.0	39.1 ± 11.5	34.1 ± 3.5	38.1 ± 5.8
Total *n-*6	4.9 ± 1.3	3.2 ± 1.3	6.5 ± 1.2	6.4 ± 0.3
Total other	4.0 ± 1.2	4.4 ± 2.8	5.2 ± 1.5	4.9 ± 1.9
**Lipids**				
TAG	1.9 ± 2.2	2.7 ± 1.7	4.9 ± 3.3	7.5 ± 5.8
ST	7.1 ± 1.6	4.7 ± 2.7	8.1 ± 1.8	6.9 ± 0.7
PL	87.8 ± 6.0	91.3 ± 2.5	86.8 ± 1.8	83.1 ± 7
**Lipid content**				
g lipid /100 g wet weight	0.8 ± 0.4	1.2 ± 0.6	1.1 ± 0.1	1.3 ± 0.2
g lipid/100 g dry weight	3.2 ± 1.7	4.6 ± 2.7	5.0 ± 0.7	5.8 ± 1

TLE: Total lipid extract; Values are mean ± standard error (*n* = 6); Other SFA: Sum of 15:0, 17:0, 20:0, 22:0, and 24:0; Other MUFA: Sum of 16:1*n-*5, 18:1*n-*7trans, 18:1*n-*5, 22:*n-*11, and 22:1*n-*9; Other PUFA: Sum of 16:2*n-*6, 18:3*n-*6, 20:4*n-*6, 20:2*n-*6, 22:5*n-*6, 22:4*n-*6, and 22:2*n-*6; Other includes fatty aldehydes and 4,8,12 trimethyl tetradecanoic acid (4,8,12-TMTD); TAG: triacylglycerols, PL: polar lipid; ST: sterols.
